# IoTactileSim: A Virtual Testbed for Tactile Industrial Internet of Things Services

**DOI:** 10.3390/s21248363

**Published:** 2021-12-15

**Authors:** Muhammad Zubair Islam, Shahzad, Rashid Ali, Amir Haider, Hyungseok Kim

**Affiliations:** School of Intelligent Mechatronics Engineering, Sejong University, Seoul 05006, Korea; zubair@sju.ac.kr (M.Z.I.); shahzad@sju.ac.kr (S.); rashidali@sejong.ac.kr (R.A.)

**Keywords:** 5G/6G, URLLC, tactile Internet, industrial IoT, network emulator, robotic simulator, virtual testbed

## Abstract

With the inclusion of tactile Internet (TI) in the industrial sector, we are at the doorstep of the tactile Industrial Internet of Things (IIoT). This provides the ability for the human operator to control and manipulate remote industrial environments in real-time. The TI use cases in IIoT demand a communication network, including ultra-low latency, ultra-high reliability, availability, and security. Additionally, the lack of the tactile IIoT testbed has made it more severe to investigate and improve the quality of services (QoS) for tactile IIoT applications. In this work, we propose a virtual testbed called IoTactileSim, that offers implementation, investigation, and management for QoS provisioning in tactile IIoT services. IoTactileSim utilizes a network emulator Mininet and robotic simulator CoppeliaSim to perform real-time haptic teleoperations in virtual and physical environments. It provides the real-time monitoring of the implemented technology parametric values, network impairments (delay, packet loss), and data flow between operator (master domain) and teleoperator (slave domain). Finally, we investigate the results of two tactile IIoT environments to prove the potential of the proposed IoTactileSim testbed.

## 1. Introduction

**Figure 1:**
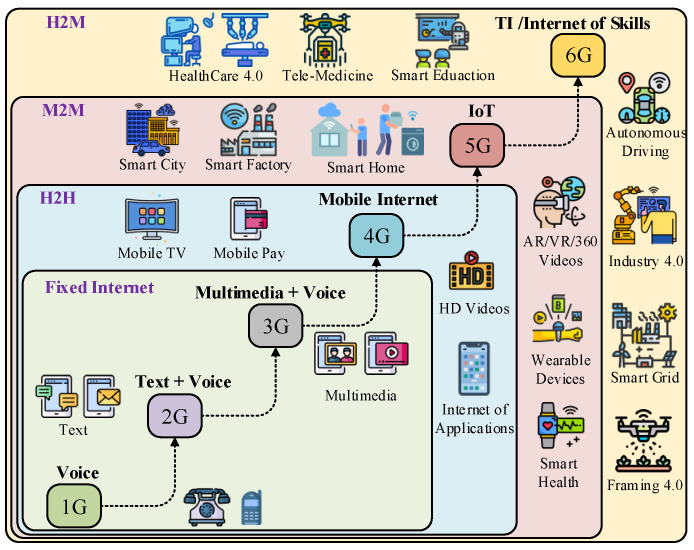
A taxonomy of the different emerging communication trends.

The rapid development of communication technologies from First-Generation (1G) to Sixth-Generation (6G) has gained enormous attention due to its emerging services like human-to-human (H2H), machine-to-machine (M2M), and human-to-machine (H2M) communication. These emerging services are induced by drivers like mobile Internet, Internet of Things (IoT), and tactile Internet (TI). The IoT envisions to fill the gap between the cyber and physical world [1]. It is defined as to interrelate every existing computing object around us such as, mobile devices, sensors, and actuators, over the Internet. Moreover, IoT technology provides data sharing and communication in the M2M environment. Recently, the TI, with the aim to enable haptic communications, has shifted the IoT paradigm to real-time interaction between H2M and revolutionized the next-generation communication technologies [2,3]. The TI is envisioned to empower H2M communication where a human can interact with machines in a virtual and physical environment, while experiencing the haptic sensations (touch and forces) in addition to traditional audio-video data [4]. Figure 1 depicts the technological evolution of the communication trends from 1G to 6G wireless communication.

Several international standard organizations, such as the international telecommunication union, the Third-Generation Partnership Project (3GPP), and the Institute of Electrical and Electronics Engineering (IEEE), are working to enable the existing and develop new network architectures to carry haptic data over the communication in real-time. The TI standard working group IEEE P1819.1 has already initiated and defined reference architecture, technical functions, and the definition of the TI [5]. Moreover, it also described standard use cases of the TI and corresponding strict requirements, including teleoperation, automotive, immersive virtual/augmented reality, internet of drones, interpersonal communication, live haptic broadcast, and cooperative automated driving. However, these use cases demand near real-time connectivity (ultra-reliable and ultra-responsive) for M2M and H2M communication. This type of real-time connectivity is termed as ultra-reliable and low latency communication (URLLC). The URLLC is one of the key services of the Fifth-Generation (5G) networks, along with enhanced mobile broadband and massive machine-type communication. Moreover, 3GPP has introduced the 5G new radio to increase reliability and minimize end-to-end (E2E) communication latency for the URLLC services. In Release 15, 3GPP describes the URLLC requirement with the reliability of 99.9% for a single 32-byte packet under 1ms latency [6]. Conclusively, 5G URLLC services are one of the potential enablers for the extreme requirements of the TI.

**Figure 2:**
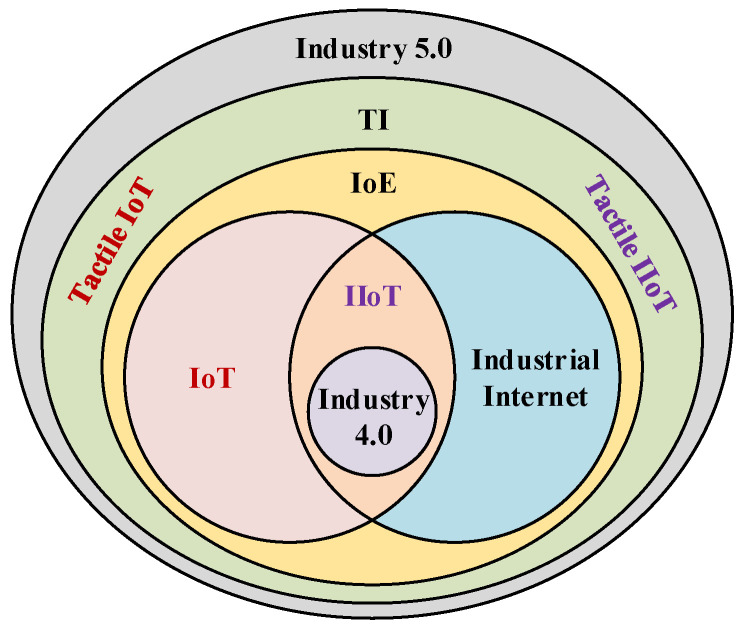
An overview of the relation between IoT, IIoT, tactial IoT, tactile IIoT, Industry 4.0, and Industry 5.0.

Moreover, these requirements become more critical for loss-intolerant and delay-sensitive TI industrial and medical applications. For example, remote industrial management and the automation of industrial robots (sensitivity of control circuits) demand latency between 0.25–10 ms with a packet loss of ≤$10^{-9}$ [7]. Therefore, supporting next-generation industrial applications, including immersive reality, holographic, and haptic/tactile communication, demands a 5G network with new physical and upper layer techniques to guarantee quality of service (QoS) and quality of experience (QoE) provisioning. Furthermore, the 6G technology paradigm promises to break the 5G network limitations and enable them to virtualize human skills and transfer them from one place to another within 1ms through 6G native artificial intelligence (AI) network architecture. In-depth work on 5G URLLC services, beyond the 5G and 6G communication network, is presented in these articles [8,9,10,11,12]. Table 1 compares the connectivity requirements of the traditional and emerging tactile IIoT applications (adapted from [7]). The relationships between emerging technologies such as IoT, IIoT, Industrial Internet, Internet of Everything (IoE), TI, tactile IoT, tactile IIoT, Industry 4.0 and 5.0 are presented in Figure 2.

An in-depth discussion on conventional and emerging industrial is presented in [7], where the authors investigated the role of TI in the industrial environment, along with technical connectivity requirements of the TI industrial services. One of the vital use cases of the TI in the industrial domain is the bilateral/multilateral haptic-driven teleoperation systems. A teleoperation system consists of a human operator (master), teleoperator (slave), and a communication network that link the master to a slave domain, and enable the operator to interact with the teleoperator in the distant and inaccessible remote environment to perform complex tasks. The TI-based network provides bilateral communication to manage touch and actuation in real-time between the master and slave domain with a focus to ensure QoS and QoE requirements. Haptic-enabled teleoperation systems have numerous applications in Industry 4.0, such as robotic automation, smart manufacturing, smart logistic, the mining industry, food industry, healthcare industry, and industrial management (controlling and monitoring). Contrary to the traditional application, Haptic-enabled industrial applications demand high QoS and QoE, and depend on the nature of the application.

One of the effective ways to investigate the tactile IIoT application requirements, performance, and testing the new solutions to ensure QoS and QoE, is to set up a virtual testbed similar to the real network. The testbed must allow us to utilize and maintain hardware and software virtually on a standard computer without purchasing them. In the literature, several recent articles have proposed testbeds to overcome the above-mentioned challenges. The work in [13] proposed a haptic system testbed to characterize and validate E2E haptic communication of different use cases of TI. The authors introduce a framework comprised of multiple sub-blocks that can be re-configured based on the nature of use cases, with a focus on minimizing cost and evaluation time. It also provides an option to integrate the testbed with the simulation platform through a connector interface to perform testing. Commonly, it is intended to offer an extensive range of haptic hardware, including sensors, actuators, and tactile interface boards. A testbed for tactile and kinesthetic data coding was proposed in [14] aligned with IEEE P1918.1 TI standard working group to improve and standardize haptic codec. The proposed haptic coding testbed is considered as a reference testbed with the aim to develop optimal data compression schemes to exchange tactile and kinesthetic information and enable human-in-the-loop TI services. The authors also provide some reference tactile data traces, software, and hardware to evaluate newly developed kinesthetic and tactile codecs.

In [15], a framework for tactile cyber physical systems was proposed, which is specifically for physical remote environments and based on network simulator NS3. It provides an interface for robotic experiments, along with haptic communication modules. However, the authors ignored the extensibility of the proposed testbed for other haptic-driven applications. Similarly, the authors in [16,17] proposed a generic testbed framework for different TI use cases. A data-driven experiment setup was proposed in [16] to provide a common playground for testing haptic applications. The proposed haptic communication testbed at the Otto-von-Guericke University of Magdeburg (OVGU-HC) focused on providing experiment testbed for long-distance haptic-enabled teleoperation systems, in addition to small scale wireless haptic-driven applications. The OVGU-HC presents experiment automation and data collection utilizing experiment description language (DES-Cript). The proposed OVGU-HC did not work standalone, is a part of the MIoT-Lab, and is just used to gather hepatic experiment information. Moreover, it utilized domain-specific language DES-Cript [18], and did not provide an open-source facility to the research and development community.

The study in [17] presents a two-level classification of the TI applications based on controlled environment and master-slave integrations to develop a generic testbed, with a focus to ensure compatibility for all these classified applications, which is named as TI- eXtensible Testbed (XT). To demonstrate the potential of the TIXT, they discuss H2M haptic communication in the virtual and physical environment. However, they ignored the explanation on how to characterize the network impairments (delay, jitter, and packet losses) and investigate the performance of the haptic-driven IIoT application. Therefore, there is a strong need for a testbed that offers flexibility, scalability, open-source availability, tailored to examine network impairments, communication flow, and extensible for TI IIoT use cases. In this regard, we proposed a virtual testbed called IoTactileSim to investigate tactile IIoT services from QoS and QoE perspectives. The IoTactileSim employs Software Define Network (SDN) and edge computing at the core network to tactile industrial application. The following section presents the main contribution of the proposed IoTactileSim testbed.

### 1.1. Research Contributions

The primary contributions of this work are summarized below as:

- We presented the details of TI in the context of various industrial environments and discussed some emerging applications of the tactile IIoT.
- A hybrid virtual testbed, IoTactileSim, is proposed by combining a network emulator and an industrial robotic simulator to simulate tactile IIoT applications and investigate their performance.
- We designed the IoTactileSim by adopting a hierarchical approach, where the network is divided into two parts; a core and an edge layer. The core layer consists of SDN routers to perform intelligent routing, while the edge layer performs as an intelligent support engine for tactile IIoT services.
- The proposed IoTactileSim identifies the challenges imposed by the tactile IIoT and their strict QoS/QoE requirements. Moreover, it focuses on investigating the communication network parameters (latency, reliability) and other configurations corresponding to the identified requirements.
- We conduct two different experiments in the tactile IIoT environment to evaluate the performance and present the potential of the proposed IoTactileSim testbed.

### 1.2. Paper Organization

**Figure 3:**
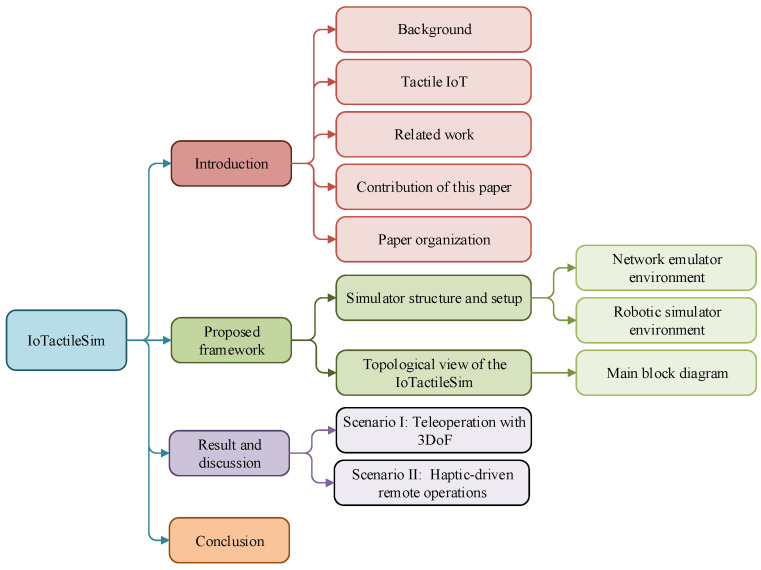
Diagrammatic view of the structure of the paper.

As illustrated in Figure 3, the rest of this paper is organized as follows. Section 2 discusses the proposed IoTactileSim structure and setup, along with the topological view. Section 3 presents the scenarios and case studies to demonstrate the potential of the proposed testbed. Finally, Section 3 concludes the paper and offers some future avenues.

## 2. Proposed Framework

In this section, we describe the proposed IoTactileSim testbed to support a broad range of tactile IIoT services. At first, we will present the network emulator to mimic the real-world communication network, followed by a detailed discussion on the industrial robotic simulator. Finally, we present the topologic view of the proposed simulator, along with the basic parametric settings.

### 2.1. Simulator Structure and Setup

**Figure 4:**
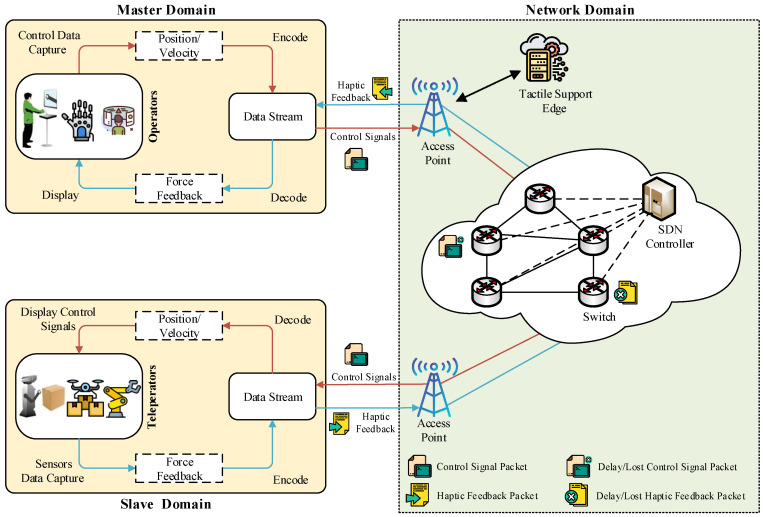
Indepth overview of the proposed virtual testbed IoTactileSim.

The structure of the proposed testbed IoTactileSim as depicted in Figure 4, following the IEEE P1918.1 TI standard architecture. In general, the TI use cases are comprised of three key domains: master domain, network domain, and slave or controlled domain [5]. The master domain consists of operators (human or control algorithms) that exploit haptic devices. The slave domain deals with the slave robots or teleoperators that the master side operator directly controls via control signals. The network domain connects the master and slave sides to enable bi-directional communication. To control the slave side teleoperator, the master side sends the control signal, and in return receives the feedback information including haptic and audio-visual signals. The master and slave domain creates a global control loop over communication network infrastructure. To maintain stability for the tactile IIoT services and provide a real-time haptic sensation to the users, this global control loop demands a haptic packet sampling rate of ≥1 kHz, a packet loss rate between $10^{-3}$–$10^{-5}$, and latency ranging from 1–10 ms. The proposed IoTactileSim helps the users to investigate these strict requirements and evaluate their newly developed strategies for emerging industrial applications.

#### 2.1.1. Network Emulator Environment

As can be seen in Figure 4, the control signal is captured through the controller on the master side and forwarded to the network domain in a specifically encoded format. SDN, network function virtualization (NFV), and mobile edge computing (MEC) are employed with 5G technology in the core network of the proposed testbed to overcome the 1 ms latency challenge and to provide support for the next-generation industrial applications [19]. The network domain receives these packets and forwards them to the slave domain to perform the required task. The feedback in form of the haptic data is sent from the slave teleoperator to the master side human operator. The proposed IoTactileSim utilizes the Mininet emulator for the network design, resembling a real-world network operations and hardware in a virtual environment [20]. Mininet employs process-level virtualization to develop a virtual communication network with virtual hosts and connects them via virtual Ethernet pairs.

**Figure 5:**
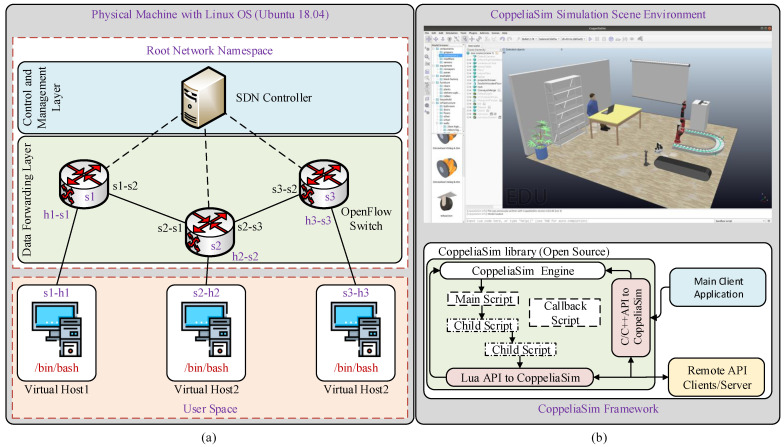
Overview of the basic framework for Mininet and CoppeliaSim. (**a**) represents the Mininet network emulator with Open vSwitch and SDN based OpenFlow protocol, (**b**) illustrates the CoppeliaSim simulator scene environment along with control architecture.

The proposed IoTactileSim enables the evaluation of large custom topologies with actual application traffic traces by deploying them into the physical network. It also enables the utilization of emerging technologies such as SDN, NFV, and MEC. In the SDN framework, control planes are separated from the forward plane in the network. The emulator is written in python language and freely available at the Mininet official website (http://mininet.org/, accessed on 10 November 2021). An overview of the basic architecture of the Mininet with open-source virtual switches Open vSwitch (OVS) and SDN standard protocol OpenFlow is depicted in Figure 5a. Mininet by itself is a network emulator that allows users to mimic real network topologies. It also enables users to build such network topologies in SDN architecture. This is what Mininet is capable of in a nutshell. It does not provide any support for integrating tactile Input/Output (I/O) modules or any other modules for that matter. It only provides us with a virtual environment where all network nodes are present (virtually) on a single physical device. The contribution of the IoTactileSim over Mininet is defined as:

- IoTactileSim allows users to integrate several tactile I/O modules with the Mininet environment.
- IoTactileSim enables users to implement each network module on a separate physical device.
- IoTactileSim also has an embedded tactile support engine which is not present in Mininet. This support engine can be modified by the user based on their use cases.

#### 2.1.2. Robotic Simulator Environment

To develop the smart industry with human-in-the-loop and human-robot interaction haptic-driven teleoperation use cases like remote maintenance, inspection, industrial management, we utilized one of the famous industrial robotic simulators CoppeliaSim. This industrial robotic simulators CoppeliaSim is formerly known as (V-REP: Virtual Robot Experimentation Platform) [21]. The reason to use the CoppeliaSim is that it provides a range of emerging industrial applications, including factory automation, remote monitoring, safety monitoring, telerobotic operations, etc. Figure 5b illustrates the basic control architecture and scene environment of the CoppeliaSim simulator. As can be seen from Figure 5b, the simulation loop consists of the main and child script. The main script controls all child scripts attached to the specific object in the simulation environment. The remote Application Programming Interface (API) allows the user to interact with the simulator from outside the system through socket communication. The remote API client and server are responsible for providing these services through different programming languages like C/C++, Python, Matlab, Java, etc.

**Figure 6:**
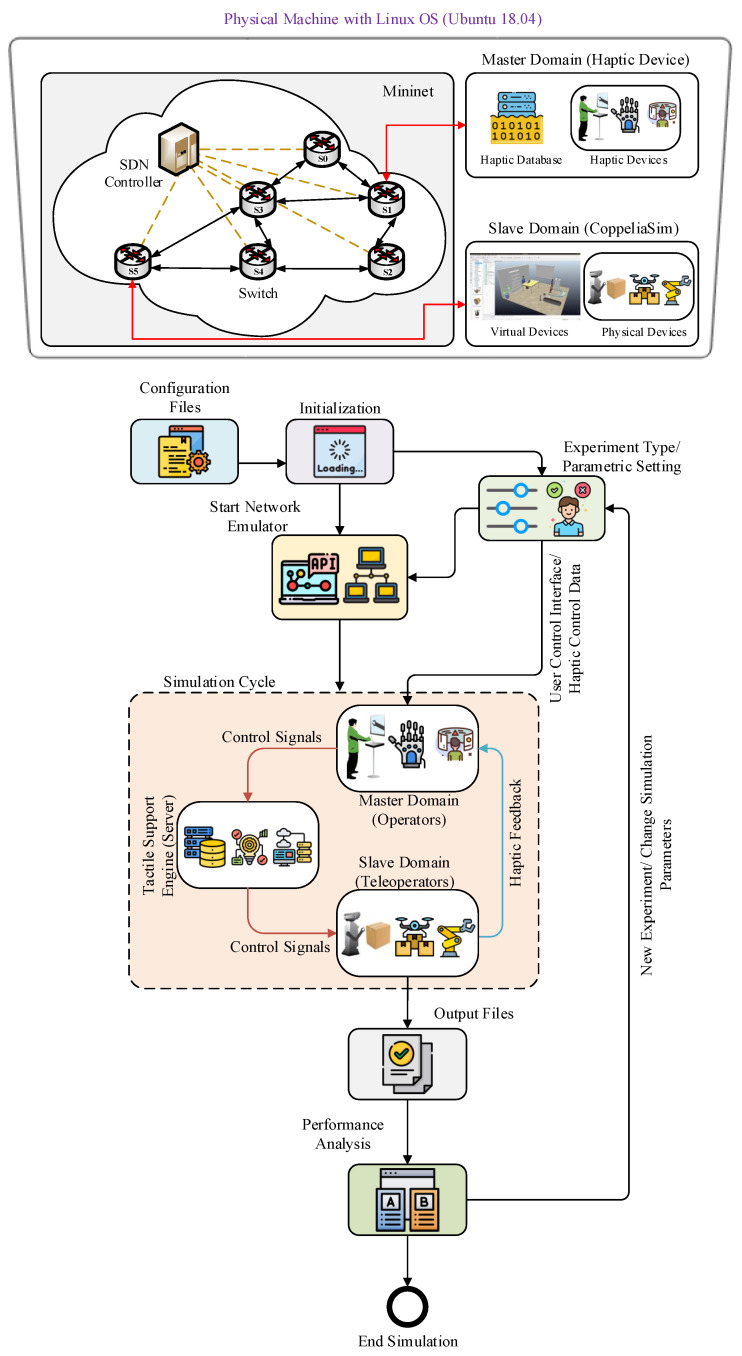
The flowchart illustrating the overall flow of the IoTactileSim testbed dynamics and relationship between different parts.

In the proposed IoTactileSim, we utilized the python remote API client to interact with the smart industrial application in the CoppeliaSim environment through socket communication. The control code of the developed IIoT applications is executed in the same computing machine where the network emulator was employed. The CoppeliaSim simulator is connected with a network to represent network-slave interaction that was designed utilizing Mininet. Additionally, the network is linked with the master domain and makes the master-network relationship. The overview of the connection between master, network, and slave domain using Mininet and CoppeliaSim in a single computing machine (personal computer) is illustrated in Figure 6, and an in-depth discussion on each module is presented in the next section.

### 2.2. Topological View of the IoTactileSim

This section focuses on the architecture of the proposed IoTactileSim testbed as depicted in Figure 6. At first, we discuss the parameter initialization, settings, and user interface to interact with the proposed testbed. Second, the topological view of the core network architecture is presented. Third, an in-depth discussion on application-agnostic design with the application and network connectivity is reported.

Initialization: In the initialization module, the simulator reads the parametric configuration files. It sets packet size, packet rate, Internet Protocol (IP) suite, IP address, link bandwidth, and link latencies. This module facilitates the users to provide the parameter settings as per their experiment need. If the user does not provide the parametric settings, then it automatically uses the default values of the parameters, as defined in Table 2.

Start Network Emulator: In order to design a real-world network, a Mininet emulator creates a custom topology with five OVSs and three hosts. The hosts act as a standard computing machine and are responsible for the master domain, slave domain, and tactile support engine. The OVSs are connected with a single SDN controller. The SDN controller decides to handle the data plane and allows the network operator to control and manage the whole network via API provided by the Mininet. Therefore, we utilized the Mininet Python API in IoTactileSim, so that the users can change the network settings as per their experiment demands to evaluate their newly developed approaches. The users can change the parametric values from the configuration files as discussed earlier in the initialization step.

Simulation Cycle: After creating the network topology with three hosts that work as a master, slave, and tactile support engine, the simulator enters into the simulation cycle. In the simulation cycle, the actual experiments are performed as per defined conditions by the users through parametric settings or default values. The network host that acts as the master side, connects to the haptic device that the human operator uses to send the control signals to the salve side manipulator. The other host acting as a slave, connects to the CoppeliaSim simulator for executing the control commands in the designed virtual/physical tactile IIoT applications. After conducting a desired control experiment, the haptic feedback is sent to the human teleoperator at the master domain. This loop runs until the simulator reaches the defined threshold values like 1000 packets, etc. Finally, at the end of the simulation cycle, the simulator stores the experimental results into a file to investigate various network impairments.

Performance Analysis: This module receives the stored experimental data file and compiles result graphs to understand the strengths and weaknesses of the conducted experiments from the QoE and QoS perspectives. After representing the experimental result in the visual form, it stores these results, and enables the users to perform new experiments or to exit the simulator. The list of the default parameter values and settings used in the proposed IoTactileSim are summarized in Table 2. The proposed IoTactileSim is publicly available at Github (https://github.com/zubair1811/IoTactileSimV1.git, accessed on 30 November 2021) to the interested researchers to conduct extensive experiments to evaluate their suggested approaches.

## 3. Result and Discussion

In this section, we demonstrate the effectiveness of the proposed IoTactileSim with two different tactile industrial scenarios using the simulation environment and parameters setting defined in Table 2. These two realistic applications belonging to tactile industrial use cases define as follows

- Scenario I: Teleoperation with 3 Degree-of-Freedom (3DoF)
- Scenario II: Haptic-driven remote operations

Moreover, these scenarios can be classified into two categories, like offline and real-time applications. On the one hand, offline (teleoperation with 3DoF) experimentation means that we already have a static dataset of some real-world teleoperation applications and utilize previously collected data for analytical analysis. On the other hand, real-time or online (haptic-driven remote operations) experiments indicate that interaction data between operator and teleoperator are collected in real-time to make a suitable decision to ensure stability and transparency. The real-time scenario is complex compared to offline because it deals with more data under time constraints. Most of the existing studies on industrial testbeds just utilized the offline methodology, while the proposed IoTactileSim considered both scenarios. The discussion considering offline and real-time scenarios is presented in detail in the following subsections.

### 3.1. Scenario I: Teleoperation with 3DoF

**Figure 7:**
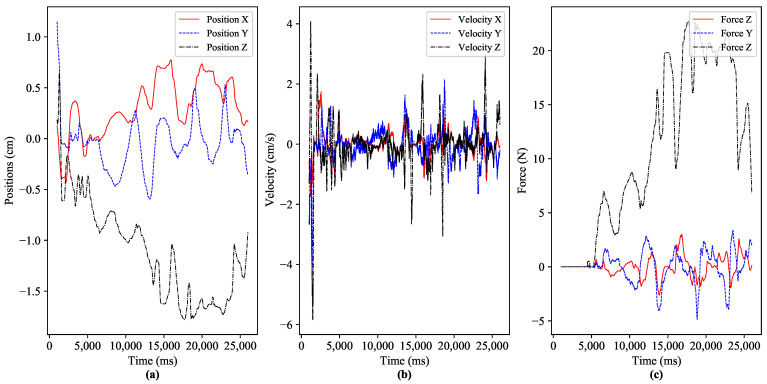
Dynamic interaction of the human operator with virtual application via haptic device. (**a**) positions of the human operator’s hand at master side device, (**b**) velocity traces of the operator (**c**) force data traces of the teleopertor x, y and z-axis.

Scenario I considers the offline experimentation, where publicly available 3DoF haptic traces in [22] on teleoperations were utilized. To record the haptic traces, a human operator employs a haptic device (Phantom Omni) at the master to interact with the virtual environment, which acts as a slave domain. The virtual environment is comprised of a rigid movable cube lying on a wooden, smooth surface. The human operator makes an interaction (static and dynamic) with the rigid cube via the haptic device and receives force feedback. Figure 7 illustrates the 3DoF position and velocity control by the human operator via the haptic device and the received force feedback of the used haptic dataset [22] for experimentation. In the proposed IoTactileSim testbed, we transmit the control signals (position/velocity) from the master side to the slave side and get the haptic feedback (force). To minimize the E2E communication delay, a common practice is to transmit the haptic traffic packets instantly after receiving sensors’ data, resembling a real-time tactile industrial IoT application.

We adopt the same method in our experiments; after reading the sensors data, the system makes packets of the haptic traces as following:

(1) $$PacketSize=Ethernet/UDP/IP/8\times N_{DoF}$$

where Ethernet/UDP/IP indicates the header of the ethernet, user datagram protocol, and internet protocol layer, respectively. $N_{DoF}$ is the number of DoF in the experimental data. We are using 3DoF, so the formulation can be evaluated as:

$$\begin{array}{c} Packet\;Size=14/8/20/8\times 3\\ Packet\;Size=14+8+20+24=78 \end{array}$$

**Figure 8:**
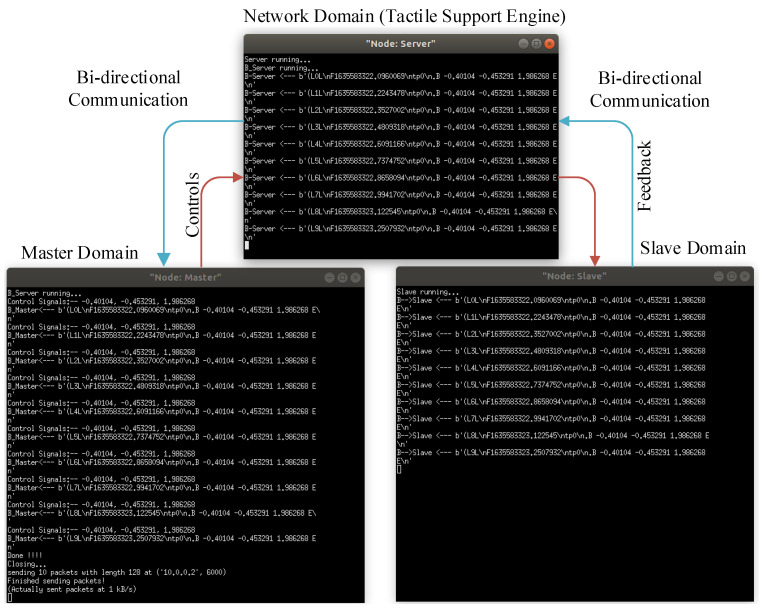
IoTactileSim interface for scenario I experiment.

The interface of the IoTactileSim during communication between master and slave domain is depicted in Figure 8. At the master domain, control signals from utilized 3DoF haptic traces are selected and transmitted to the slave domain through the network domain. Similarly, after receiving specific control signals, the slave domain returns the corresponding force feedback to the master domain. The overview of the data flow interfaces between master and slave for the scenario *I* is depicted in Figure 8.

**Figure 9:**
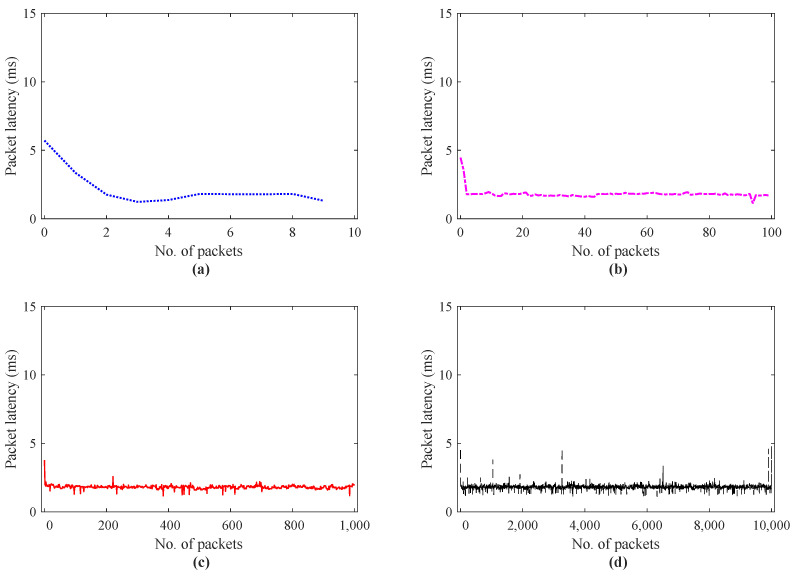
Packet latency investigation for scenario I haptic data transmission; (**a**) data packets = 10, (**b**) data packets = 100, (**c**) data packets = 1000, (**d**) data packets = 10,000.

**Figure 10:**
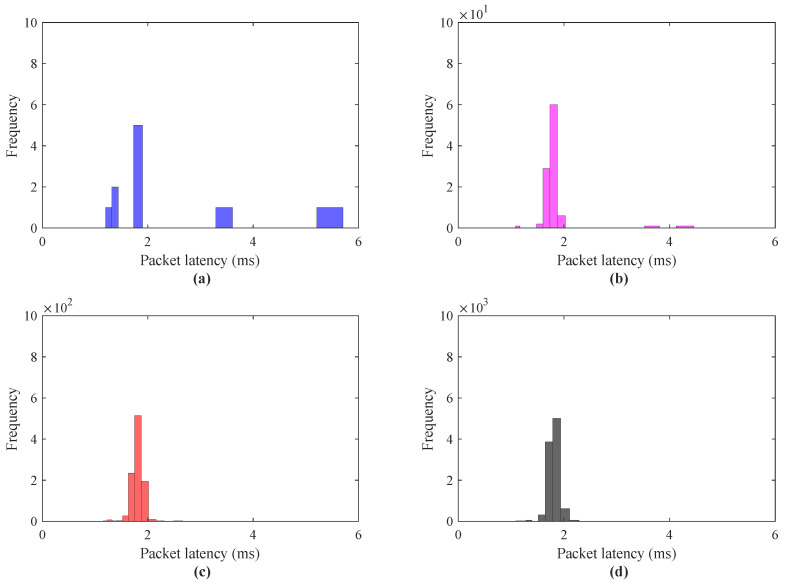
Packet latency histogram for scenario I haptic data transmission; (**a**) data packets = 10, (**b**) data packets = 100, (**c**) data packets = 1000, (**d**) data packets = 10,000.

The performance analysis for a scenario I in terms of round trip delay is presented in Figure 9. To investigate the effect of the number of haptic data packets on round trip delay for IoT applications, the scenario I was simulated for the number of haptic data traces = 10 to 10,000. The latency investigation using IoTactileSim for a scenario I with the number of packets = 10, 100, 1000, 10,000 is depicted in Figure 9a–d, respectively. It can be seen clearly from the results, the tendency with an increase in the number of haptic data packets the round trip delay decreases from 5 to 2 ms. In Figure 9a, with the number of packet = 1, the packet latency approaches 6 ms as compared to Figure 9b–d, where packet latency is below 5 ms. To elaborate this latency decrement in detail, Figure 10 illustrates the packet delay histogram for a scenario I.

Contrary to Figure 9, the results in Figure 10 reveal the latencies of the most frequent haptic data packets. Similar to the results presented in Figure 10a–d, the simulation results in Figure 10a–d also indicate the decrease in packet latencies from 5.8 to 2.1 ms. From Figure 10a–d, it can be seen clearly that most of the haptic traces latencies centered between 1 to 2 ms, which is one of the stringent requirements for the tactile IoT services. Figure 10b–d, depicts the improvement in the communications network impairments (delay, jitter) with the number of packets = 100, 1000, 10,000. The reason behind this is that at the beginning, the proposed testbed IoTactileSim understands and fine-tunes the simulation parameters to support delay-sensitive and loss-intolerant applications. The efficacy of the scenario I regarding reliability characterization is summarized in Table 3. The reliability of the transmitted haptic data packets is evaluated in terms of delayed/lost and out-of-ordered packets.

### 3.2. Scenario II: Haptic-Driven Remote Operations

**Figure 11:**
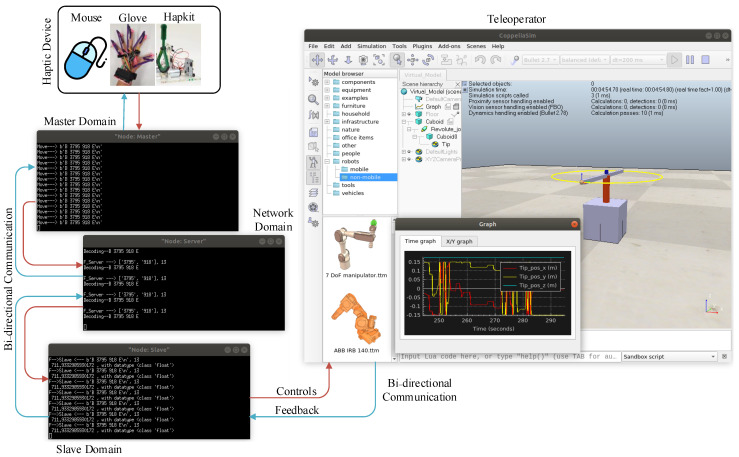
IoTactileSim interface for scenario II experiment.

In this section, we will present the real-time control of the teleoperator in the virtual environment to mimic the real-world tactile industrial remote operations. Similar to the scenario I, II also consists of the master, slave, and network domain where virtual teleoperator developed in CoppeliaSim acts as salve domain. In the master domain, physical haptic devices (haptic computer mouse, glove, and hapkit) are used to interact with the virtual environment, as illustrated in Figure 11. These haptic devices are easy to develop because their supplementary material is available publicly for the research community. The tactile computer mouse was presented in [14], while the study in [23,24] provide the design and development detail on a haptic glove and Hapkit, respectively. However, in this experiment, we only employed the (computer mouse and glove) to interact with the teleoperator as slave side. We mapped the physical computer mouse and glove X and Y direction to the XY coordinates of the developed virtual teleoperator in CoppeliaSim. The key focus of this experiment is to investigate the communication network parameters (latency, reliability) that affect the TI services. Additionally, it also demonstrates the potential of the proposed IoTactileSim to provide TI services under TI QoS/QoE requirements (1–5 ms). Human operators in the master domain use the haptic device to interact with the teleoperator at the slave side and receive haptic feedback. The interface of the IoTactileSim during direct controlling of the teleoperator in the virtual environment is depicted in Figure 11.

**Figure 12:**
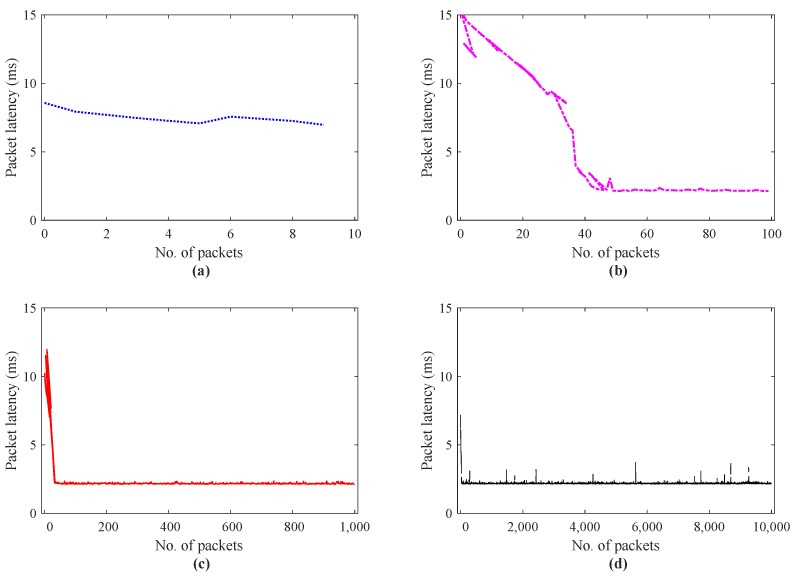
Packet latency investigation for scenario II real time haptic-driven remote operation; (**a**) data packets = 10, (**b**) data packets = 100, (**c**) data packets = 1000, (**d**) data packets = 10,000.

To observe the effect of the number of data packets on latency for scenario II, the simulation results are summarized in Figure 12. These results also demonstrate that with the increase in the number of packets from 10 to 10,000, the network communication latency tends to decrease. In this experiment, we directly control the teleoperator in the virtual environment via a computer mouse in real-time. The control signals from the computer mouse are packetized as defined in (1), sampled as per haptic system requirement, and transmitted to the teleoperator using default parameters values as listed in Table 2. The teleoperator at slave side receives the control commands to perform the required task and backward the force feedback to the human operator. Figure 12a–d, indicates the results of the packet latency for the number of data packets 10, 100, 1000, 10,000, respectively. In Figure 12a with the number of packets = 10, the value of latency lies between 9–7 ms.

In Figure 12b, up to 40 packets, latency value remains higher than 5 ms, and after that, the system gets convergence round trip latency around 2.5 ms. Similarly, the results in Figure 12c, gain a minimum latency value of 2.5 ms from the 20th data packet to the 1000th packet. To continue on a similar line as mentioned above, Figure 12d, exhibits a quick decreasing trend in round trip latency from 6 to 2.2∼2.0 ms, as the number of data packets increases from 10, to 10,000.

**Figure 13:**
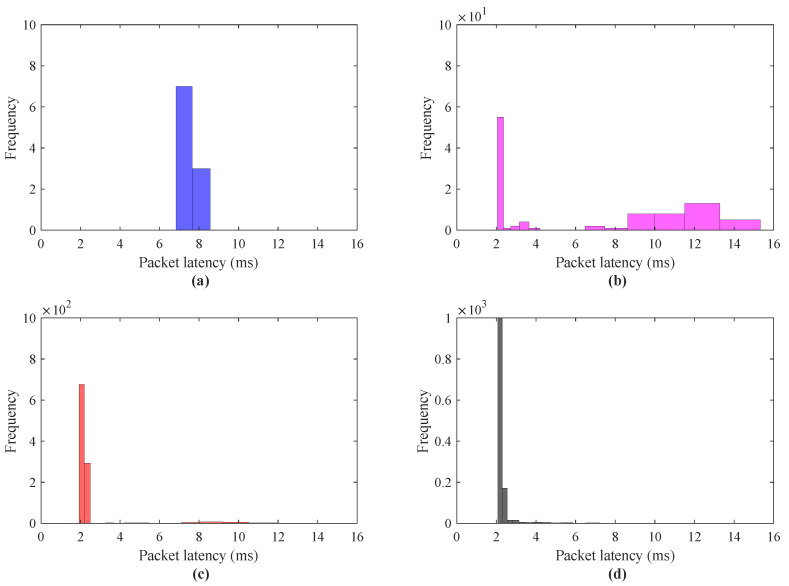
Packet latency histogram for scenario II real time haptic-driven remote operation; (**a**) data packets = 10, (**b**) data packets = 100, (**c**) data packets = 1000, (**d**) data packets = 10,000.

To elaborate this packet latency convergence in a better way, Figure 13 illustrates the histogram of the frequent data packets regarding packet latencies. The results in Figure 13c,d indicate that the packet latency reduces for an increase in the number of packets compared to results in Figure 10c,d. In addition, these results depict that the latency is more concentrated between 2 to 2.3 ms. It is also interesting to observe that, for the higher number of the data packet with a higher sampling rate, the proposed IoTactileSim is capable of reducing congestion and maintaining the application latency requirement. In addition Table 3 presents the in-depth reliability analysis for the scenario II experiment. As it can be seen clearly from Table 3 for scenario II the percentage of delayed or dropped packets decrease from 100% to 0.40% (10 to 39 data packets) with 100% to 0.30% out-of-order sending packets from 10 to 10,000 number of data packets.

In summary, the packet latency convergence analysis in Figure 9 and Figure 12 and periodic packet variation analysis Figure 10 and Figure 13 for use case scenarios I and II indicates that the proposed virtual testbed IoTactileSim provides the facility to the users to implement complex tactile industrial use cases, evaluate their proposed strategy, and investigate the QoS and QoE requirement of the implemented tactile IoT services. Some of the complex tactile IIoT use cases are illustrated in Table 4. The proposed IoTactileSim concentrate on providing QoS and QoE provisioning by taking different network parameters into account. Based on the mentioned complex tactile IIoT use cases along with requirement specification (delay, packet size, packet rate, packet loss rate, etc.) in Table 4 it indicates that IoTactileSim can ensure strict QoS-based traffic. The main objective of this paper is to provide a tool to minimize the network development cost while realizing the stringent QoS/QoE requirements for tactile IIoT applications. Moreover, it also offers to implement edge intelligence to the designed tactile support engine, which can be leveraged to improve QoS and QoE provisioning in highly dynamic network environments. The users can deploy machine learning, specifically reinforcement learning models, to track the frequently dynamic network environment states and make online decisions to improve network conditions and support time-varying user demands.

## 4. Discussion and Future Work Directions

**Figure 14:**
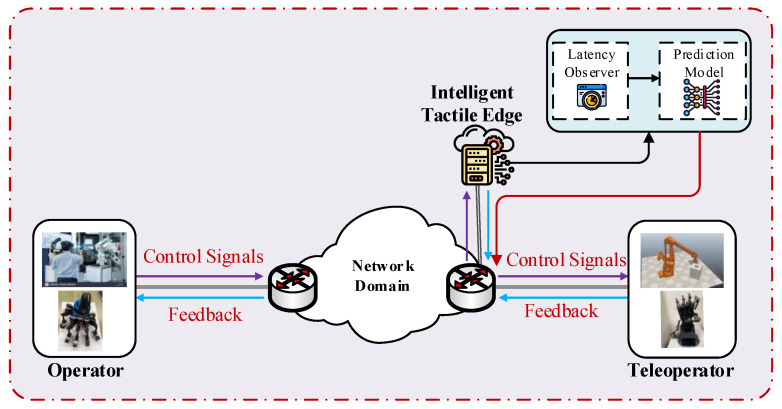
Conceptual architecture of ITE in IoTactileSim.

In our previous work [23], we analyzed the different haptic gloves and investigated how data processing increased the latency in the haptic communication loop and proposed a low-latency haptic open glove (LLHOG). Contrary to previous work, the focus of this paper is to provide network infrastructure to transmit haptic traffic between operator and teleoperator and simulate delay-sensitive and loss-intolerant tactile IIoT applications. However, there are various industry 4.0 applications under use cases class C, such as fleet management, tactile-driven logistics, cooperative robotics, and motion control, which demand higher QoS and QoE. To allow these real-time applications, the utilization of edge computing is required. Therefore, there is a need for edge-based network systems with native machine learning parts to provide the QoS and QoE requirement provisioning for these applications. In this regard, as a future, an edge-based ITE is developed as a tactile support engine to enable the ability for the user to train and deploy machine learning models at the edge to ensure QoS and QoE. The conceptual diagram to design and deploy the trained model on ITE is illustrated in Figure 14.

**Figure 15:**
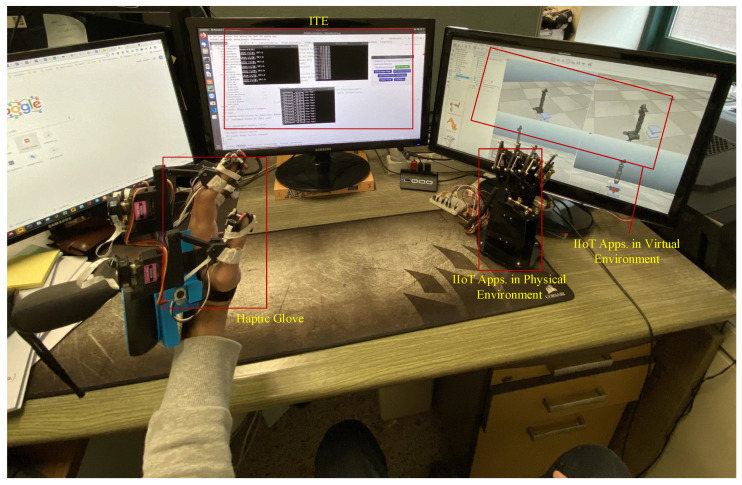
Modular representation of ITE in real-world scenario.

In future work, more practical challenges regarding tactile IIoT in the real-world scenario need to be considered. As discussed above, providing required QoS and QoE in the real-time complex industrial application is more challenging than simulation analysis. Therefore, we indented to test the proposed IoTactileSim in real-time physical IIoT scenarios and demonstrate the real-world experiment design overview in Figure 15. On the master side, we utilized the LLHOG, which consists of the rotary position sensors with a min-max scaling filter to send haptic data. The bionic robot hand, which consists of Arduino and servo controllers, is used at the slave side. The specification, sample code, and documentation are available at the official website (https://wiki.dfrobot.com/, accessed on 30 November 2021 ). The proposed IoTactileSim connects the LLHOG and bionic robot hand to develop a closed control loop. The ITE is also integrated with the proposed IoTactileSim to monitor the network dynamics and guarantee the QoS and QoE requirements for tactile IIoT applications.

## 5. Conclusions

In this paper, we proposed a virtual testbed termed as IoTactileSim to investigate and provision QoS and QoE strict requirements for tactile industrial IoT applications. The proposed IoTactileSim is equipped with a network emulator Mininet and an industrial simulator CoppeliaSim to mimic the real-world communication network and industrial IoT environment. It provides the users to evaluate the efficacy of their designed strategies under possible settings, including advanced core network technologies (SND, NVF), edge intelligence, and application-agnostic parameters (packet size, sampling rate, etc.) for improving QoS and QoE. The proposed IoTactileSim is investigated for two different industrial use case scenarios with haptic data traces and real-time remote interaction. The simulation results indicate that the IoTactileSim is able to handle real-time data traffic then offline scenario by providing communication latency ranges from 6 to 2.2∼2.0 ms, and from 5.8 to 2.1 ms for 10 to 10,000 data packets, respectively. Moreover, the experimentation analysis indicates that the IoTactileSim allows the user to investigate network impairments (latency, jitter, reliability) and can support complex tactile industrial environments with a higher number of data packets. In the early future, we plan to extend the IoTactileSim with network coding and machine learning approaches like federated reinforcement learning at the tactile support engine to integrate it with the 6G network infrastructure.

## Figures and Tables

**Table 1:** Summary of the connectivity requirements for traditional IIoT and emerging tactile IIoT services.

Applications/Requirements		Latency (ms)	Reliability (%)	Scalability	Data Rate (Mbps)
Conventional	Monitoring	50–100	99.9–99.99	100–1000	0.1–0.5
	Safety control	10	99.99–99.999	10–20	0.1–1
	Motion control	0.5–2	99.9999–99.99999	10–50	1–5
	Closed-loop control	100–150	99.99–99.999	100–150	1–5
Emerging	Remote monitoring and maintenance	20–50	99.99–99.999	500–1000	1–2
	Remote operation (teleoperations)	2–10	99.999–99.99999	1–5	100–200
	Mobile workforce	5–10	99.999–99.9999	50–100	10–50
	Augmented reality	10	99.99–99.999	10–20	500–1000

**Table 2:** Summary of parameters and settings used for Simulation.

Parameters	Settings Used
Simulation environment	
Operation system	Linux (Ubuntu 18.04)
Programming language	Python 3.8
Network emulator	Mininet 3.6.9
Industrial robotic simulator	CoppeliaSim 4.2
Network emulator	
Network topology	Mesh network of switches
IP suite	User datagram protocol
Software switch type	Open vSwitch 2.9.8
SDN controller	OVS-controller
Interface protocol for controller	OpenFlow
Link latency	Shortest route 1.2 ms
	Longest route 1.8 ms
Link bandwidth	100 Mbps
No. of packets	10, 100, 1000, 10,000
Packet sampling Rate	1 kHz
Industrial robotic simulator	
Remote API	Python legacy remote API client
Simulation mode	Real-time simulation
Execution techniques	Same machine with the same thread
Interaction network	Socket communication
Simulation scene model	Custom design (Teleoperation)

**Table 3:** Summary of the reliability characterization for haptic datset and real-time haptic drive teleoperation experiment.

Experiments		Packet Statistics	
		Dropped/Delayed (%)	Out-of-Order (%)
Haptic data transmission			
Data Packets	10	20.0 (2 Packets)	11.1
	100	2.00 (2 Packets)	1.00
	1000	0.10 (1 Packets)	0.00
	10,000	0.10 (7 Packets)	0.00
Haptic-driven remote operations			
Data Packets	10	100 (10 Packets)	100
	100	44.0 (44 Packets)	44.4
	1000	3.10 (31 Packets)	3.00
	10,000	0.40 (39 Packets)	0.30

**Table 4:** Tactile IIoT use cases requirements specifications and characteristics supporting by proposed IoTactileSim.

Applications	IIoT Use Cases and Requirements				
	Cycle Time	Message Size	Data Rate	Latency	Packet Loss Rate
Control loop motion control					
Machine tools	0.5∼2 ms	20∼50 Bytes	1∼10 Mbps	0.25∼1 ms	$10^{-9}$∼$10^{-8}$
Packaging machines					
Printing machines					
Remote control					
Process automation	≤50 ms	≥10 Mbps	1∼100 Mbps	≤50 ms	≤$10^{-7}$
Process monitoring					
Process maintenance					
Fault reporting					

## Data Availability

The 3DoF haptic datasets used in this work are available online with open access for academic research use. The static and dynamic interaction haptic dataset is available online at https://cloud.lmt.ei.tum.de/s/4FmHUCsoUvwRle3 (accessed on 1 November 2021), and the simulation experiment results files are availabe at https://github.com/zubair1811/IoTactileSimV1.git, (accessed on 30 November 2021).

## Abbreviations

TI	Tactile Internet
H2H	Human-to-Human
M2M	Machine-to-Machine
M2H	Machine-to-Human
IoT	Internet of Things
IIoT	Industrial Internet of Things
URLLC	Ultra Reliable and Low Latency Communication
QoS	Quality of Service
QoE	Quality of Experience
E2E	End-to-End
SDN	Software Define Network
NFV	Network Function Virtualization
MEC	Mobile Edge Computing
OVS	Open vSwitch
